# A Comprehensive Review on Current Advances in Peptide Drug Development and Design

**DOI:** 10.3390/ijms20102383

**Published:** 2019-05-14

**Authors:** Andy Chi-Lung Lee, Janelle Louise Harris, Kum Kum Khanna, Ji-Hong Hong

**Affiliations:** 1 QIMR Berghofer Medical Research Institute, Brisbane, QLD 4006, Australia; andy.lee@qimrberghofer.edu.au (A.C.-L.L.); Janelle.Hancock@qimrberghofer.edu.au (J.L.H.); KumKum.Khanna@qimrberghofer.edu.au (K.K.K.); 2 Radiation Biology Research Center, Institute for Radiological Research, Chang Gung Memorial Hospital, Chang Gung University, Taoyuan 333, Taiwan; 3 Department of Radiation Oncology, Chang Gung Memorial Hospital, Linkou 333, Taiwan

**Keywords:** binding site, docking, Interface, modeling, peptide, peptide–protein interaction, protein–protein interaction, scoring

## Abstract

Protein–protein interactions (PPIs) execute many fundamental cellular functions and have served as prime drug targets over the last two decades. Interfering intracellular PPIs with small molecules has been extremely difficult for larger or flat binding sites, as antibodies cannot cross the cell membrane to reach such target sites. In recent years, peptides smaller size and balance of conformational rigidity and flexibility have made them promising candidates for targeting challenging binding interfaces with satisfactory binding affinity and specificity. Deciphering and characterizing peptide–protein recognition mechanisms is thus central for the invention of peptide-based strategies to interfere with endogenous protein interactions, or improvement of the binding affinity and specificity of existing approaches. Importantly, a variety of computation-aided rational designs for peptide therapeutics have been developed, which aim to deliver comprehensive docking for peptide–protein interaction interfaces. Over 60 peptides have been approved and administrated globally in clinics. Despite this, advances in various docking models are only on the merge of making their contribution to peptide drug development. In this review, we provide (i) a holistic overview of peptide drug development and the fundamental technologies utilized to date, and (ii) an updated review on key developments of computational modeling of peptide–protein interactions (PepPIs) with an aim to assist experimental biologists exploit suitable docking methods to advance peptide interfering strategies against PPIs.

## 1. Introduction

Delivering drugs specifically to patient neoplasms is a major and ongoing clinical challenge. Function-blocking monoclonal antibodies were first proposed as cancer therapies nearly four decades ago. The large size of these molecules hindered their commercial development so that the first antibody or antibody-fragment therapies were only commercialized for cancer therapeutics and diagnostics 20 years later [1,2]. A classic development during this period, a radiolabelled peptide analog of somatostatin (SST) was used to target neuroendocrine tumors expressing the SST receptor instead of targeting the receptor with an antibody [3]. The concept of using a peptide as a targeting moiety for cancer diagnosis and treatment has since led to current peptide drug developments in both academia and pharmaceutical industries. In addition to cancer treatments, peptides that mimic natural peptide hormones also offer therapeutic opportunities. Synthetic human insulin, for instance, has been long exemplified for its clinical efficacy for diabetic patients [4].

In comparison to small molecules, such as proteins and antibodies, peptides indeed represent a unique class of pharmaceutical compounds attributed to their distinct biochemical and therapeutic characteristics. In addition to peptide-based natural hormone analogs, peptides have been developed as drug candidates to disrupt protein–protein interactions (PPIs) and target or inhibit intracellular molecules such as receptor tyrosine kinases [5,6]. These strategies have turned peptide therapeutics into a leading industry with nearly 20 new peptide-based clinical trials annually. In fact, there are currently more than 400 peptide drugs that are under global clinical developments with over 60 already approved for clinical use in the United States, Europe and Japan.

Protein–protein interactions (PPIs) are the foundation of essentially all cellular process. Those biochemical processes are often comprised of activated receptors that indirectly or directly regulate a series of enzymatic activities from ion transportation, transcription of nucleic acids and various post-translational modifications of translated proteins [7]. Drugs that bind specifically to such receptors can act as agonists or antagonists, with downstream consequences on cellular behavior. Peptides and small molecules that interfere with PPIs are thus in high demand as therapeutic agents in pharmaceutical industries due to their potential to modulate disease-associated protein interactions. Accumulating evidence has suggested that better identification of targetable disease-associated PPIs and optimization of peptide drug binding characteristics will be key factors for their clinical success [8].

Unfortunately, understanding the molecular recognition mechanism and delineating binding affinity for PPIs is a complex challenge for both computational biologists and protein biochemists. This is largely because small molecules are superior in binding to deep folding pockets of proteins instead of the larger, flat and hydrophobic binding interfaces that are commonly present at PPI complex interfaces [9]. Although monoclonal antibodies are more effective at recognizing those PPI interfaces, they cannot penetrate the cell membrane to reach and recognize intracellular targets. In recent years, peptides with balanced conformational flexibility and binding affinity that are up to five times larger than small molecule drugs have attracted enormous attention [10,11]. Cyclic peptides, for example have small molecule drug properties like long in vivo stability, while maintaining robust antibody-like binding affinity and minimal toxicity [12]. In this review, we will focus two aspects of peptide drug development: (i) Fundamental technologies utilized for peptide drug developments to date, and (ii) key developments of computational modeling techniques in peptide–protein interactions (PepPIs). Recent topics and basics in conventional docking of PPIs will also be covered with an aim to assist experimental biologists exploiting suitable docking methods to advance peptide interfering strategies against PPIs.

## 2. Key Aspects in Bioactive Peptide Drug Development

### 2.1. Historic Overview

Since the first isolation and commercialization of insulin, a 51 amino acid peptide in the early 1920s, peptide drugs have greatly reshaped our modern pharmaceutical industry [13]. With advances in DNA recombination and protein purification technologies, human recombinant insulin has replaced the animal tissue-derived insulin product that was on the market for nearly 90 years. Over the past two decades, nearly 30 more peptide drugs have been approved, with over 60 in total approved worldwide. Breaking down the intended usage of these approved peptide drugs, it appears that metabolic disorders and cancer are the most predominantly targeted disease categories. A global industry analysis on peptide therapeutics predicted a compound annual growth rate (CAGR) of 9.1% from 2016 to 2024 and sales of peptide drugs to exceed 70 billion USD in 2019 [14]. The healthy growth in this industry, however, is likely attributed to the expected increasing incidence of metabolic disorders and cancers. The top selling peptide drugs for metabolic disease such as liraglutide (Victoza) and glucagon-like peptide 1 (GLP-1) both had at least two billion USD sales per annum. Popular peptide drugs including leuprolide (Lupron), gosarelin (Zoladex) and somatostatin analogs, octreotide and lanreotide also contributed to over four billion USD in sales.

### 2.2. Overcoming Intrinsic Drawbacks of Peptide Drugs

Unlike peptide drugs that are usually synthetically made, natural polypeptides such as hormones, growth factors or neurotransmitters are known to play central roles in normal physiology. Peptide drugs have two major drawbacks: Their in vivo instability and membrane impermeability [15]. Proteolytic degradation of peptide drugs in serum limits the drug’s half-life and reduces the bioavailable concentration. Routine dosing may be needed to maintain the drug at a clinically effective concentration. A number of chemical modification methods have been utilized to prevent proteolytic degradation and improve the in vivo half-life of peptide drugs. The section below gives a guide on modern technologies commonly applied to make peptides less susceptible to proteolysis.

#### 2.2.1. Termini Protection

Proteolysis can potentially take place from both N- and C-termini of a peptide by up to 500 proteases and peptidases including serum aminopeptidases and carboxypeptidases [16]. It is well-documented that different amino acid residues at N- or C-terminal will result in different degrees of proteolysis and degradation. For example, residues such as Met, Ser, Ala, Thr, Val and Gly at N-terminus are more resistant to degradation in plasma, whereas peptides rich in Pro, Glu, Ser and Thr are more susceptible [17]. If the N- or C-terminal sequences can be modified while maintaining the required targeting specificity and affinity, such modification can reduce proteolytic degradation and improve bioavailability [18]. Similarly, as long as such modifications permit proper functioning of the drug, C-terminal amidation or N-terminal acetylation can also be used to improve in vivo stability [19]. Modification with unnatural amino acid analogs may also serve the same purpose.

#### 2.2.2. Non-Chemical Methods: Identifying Critical Residues

For biologists, the choice of chemical modification often requires expertise in chemistry through collaborations. There are, however, a number of methods that can be approached easily and yet are important for biological study of peptide drug design. First, it is necessary to identify the minimum amino acid residue(s) essential for peptide activity. This can be achieved by repetitive truncating amino acids from either the N- or C-terminus of a lead sequence to ascertain the critical core peptide motif needed for biological activity. Secondly, a classic screening method called alanine scanning can be used to determine the contribution of individual amino acids to biological activity of the peptide [20]. By screening the biological functionality of a library of peptides where individual amino acids have been substituted with alanine, it is possible to identify critical amino acids. Alanine is used for the substitution because its small, uncharged side chain doesn’t interfere with the function of adjacent side chains [21]. More recently, more complex scanning techniques that take enantiomers of amino acid or further physical attributes such as acidity, basicity and hydrophobicity into account have been developed. These scanning techniques nonetheless still require validation by molecular biology and in silica methods such as mutagenesis, stability and pharmacokinetic (PK) experiments for mature development of improved biological activity. Such structure–activity relationship (SAR) studies will lead to identification of proteolytically-labile amino acids within a peptide sequence. Detailed updates on those scanning methods are not within the scope of this review and are reviewed elsewhere [22].

#### 2.2.3. Synthetic Amino Acid Substitution and Backbone Modification

The amino acid scanning techniques discussed above also provide useful information for the design of further modifications, especially on the side chain group of a particular residue. Synthetic enantiomer amino acids, for instance, have been proposed to enhance resistance to proteases as their stereochemically reversed side-chains are not recognized as protease substrates [23]. Arginine, in particular, can be effectively replaced by β3 analogues or other variants such as homoarginine, lysine or ornithine [24]. Each natural amino acid has at least a few close analogues that can be used for effective substitution on the critical residues in order to modify the rigidity and conformation of the peptide. Unnatural analogues are also available for aromatic amino acids and used to replace β-methyl groups from the heterocycles to enhance proteolytic resistance [25]. A recent preclinical success utilized side-chain modification of a momomeric helical peptide, which then acted as a triagonist to simultaneously activate the GLP-1, glucose-dependent insulinotropic polypeptide (GIP), and glucagon receptors. In a rodent model of obesity this triagonist peptide resulted in significantly reduced body weight and reduced diabetic complications without cross-reactivity at non-specific receptors [26].

Although enantiomer amino acid (D-amino acid) substitution has been a common approach to protect peptides from protease degradation, the conformational changes created can compromise the biological activity of the original L-peptide [27]. In addition to the use of D-amino acids, α-methylation and N-methylation have also been widely used. While α-methylation of amino acid provides the advantage of maintaining the side-chain at its original spatial orientation, which is critical for helical peptides; N-methylation has been proven beneficial for enhancing solubility and reducing undesired polymerization [27,28]. Such side-chain functionality-modifying strategies have evolved peptide secondary structures and yielded new peptidic molecules that are referred to as peptidomimetics. Further information on the chemistry and applications of β-/D-amino acids, α-/N-methylations, or backbone-modified semicarbazide-peptides, peptoids and peptidomimetics can be found in these reviews [29,30,31,32,33].

The protease resistance of peptides can also be improved by peptide cyclization. There are a number or strategies for generating cyclized peptides. These include head-to-tail cyclization, which generates a peptide bond between the original N- and C- termini. The amino group on lysine side-chains can be reacted with aspartic or glutamic acid side chains or the free C-terminus, forming an amide bond. Alternatively cysteine pairs can be reacted to form a disulfide bond between the side chains. These strategies can protect termini and restrict conformational flexibility of peptides, maintaining them in their bioactive conformation [34]. Cyclization between side-chains, in particular, has been proven effective in optimizing conformational stability for helical peptides. The cyclized peptide drug ATSP-7041 is an example of a recent success [35]. This side-chain cyclized α-helical (stapled) peptide is able to selectively bind to and inhibit MDM2/MDMX, thus activating p53-dependent tumor suppression [6]. Such strategies of targeting PPIs are of great therapeutic potentials as there has been vast amount of information available on disease-related PPIs in the literature but peptide-based inhibitors are only emerging to mature in the past decade.

#### 2.2.4. Computational Methods for Improving Aqueous Solubility and Membrane Permeability

The generally poor cell membrane permeability of peptides has limited their application against inaccessible intracellular targets. Peptide therapeutic development has largely focused on extracellular targets due to this limitation. Improvement of membrane permeability or development of strategies that facilitate active intracellular uptake will be critical for successful peptide-based targeting of intracellular PPIs. Potential strategies for improving intracellular uptake include modulation of the hydrophobicity and electrostatic charges to improve passive uptake, or conjugation of the active drug peptide to a cell-penetrating peptide (CPP) to facilitate its active transport.

Bioavailability of peptide biotherapeutics is usually greatly enhanced when they are more water soluble, as effective serum concentrations can be easily maintained. Optimization of aqueous solubility is however a largely empirical process, with experimental identification of unnecessary hydrophobic amino acids which can be replaced with charged or polar residues to modulate the pI while maintaining bioactivity [36,37]. Recently, two support-vector machine (SVM) machine-learning bioinformatic tools were developed to expedite this process [38]. ccSOL omics offers a large-scale calculation of solubility for not only a proteome-wide prediction, but also the identification of soluble motifs within any given amino acid sequence [39]. PROSO II is another SVM learning based online tool to predict solubility based on physiochemical properties of the primary sequence such as the degree of hydrophobicity, hydrophilicity and secondary structural propensities in coil, helix or sheet [40].

#### 2.2.5. Membrane Protein-Facilitated Intracellular Peptide Uptake

G-protein coupled receptors (GPCRs) are a superfamily of transmembrane receptors, which are responsible for transporting diverse molecules across membranes. Although peptides can be natural ligands for GPCRs, only a small subset of extracellular peptides are actively transported across the plasma membrane. These peptides capable of crossing cell membranes are now termed cell permeable peptides (CPPs). They are generally highly hydrophobic, five to 30 amino acid long peptides [41]. The molecular and structural mechanisms underlying CPP intracellular transport remain unclear, investigating CPPs towards an ultimate goal of developing peptide drugs that are cell-permeable and orally bioavailable has been an intense field of research [42]. The ability of CPPs to cross the membrane lipid bilayer has been the foundation of significant developments in biotherapeutic peptides. The highly amphipathic and cationic characteristics of antimicrobial peptides (AMPs), for example, have allowed them to penetrate cell membranes to eliminate microbes and infectives by modulating immune responses [43]. CPPs have also been exploited as targeting moieties by conjugation to deliver cargos including small molecules, peptides, proteins or antibodies that would otherwise be membrane-impermeable [44]. The covalent conjugation of a HIV TAT peptide or more recently an amphipathic cyclic peptide was demonstrated to facilitate escape from early endocytosis and effective cytosolic delivery of otherwise membrane-impermeable peptides to target PPIs [45]. There are a number of powerful bioinformatic tools that allow users to predict and optimize their experimental designs of CPPs. CPPpred web servers such CPPpred-RF or KELM-CPPpred allow the prediction and design of CPPs from a query input protein sequence using machine learning-based models [46,47,48]. CellPPD is another free webserver that provides a permeability prediction based on physiochemical properties such as hydrophobicity, amphipathicity, steric hindrance, charge and molecular weight [49,50]. Despite the lack of physiochemical analyses, CPPpred-RF and KELM-CPPpred use selected databases for CPP uptake efficiency prediction and robust CPP/non-CPP prediction, respectively. CPPsite 2.0 is a repository currently containing 1855 unique experimentally validated CPPs along with their secondary and tertiary structures. This provides an informative resource to assist web-lab researchers to design more optimal CPPs prior to labor- and time-expensive experiments [51]. Table 1 provides an overview to these online tools for analysis of peptide solubility, prediction and design of CPPs.

As discussed above, cyclic peptides have superior proteolytic resistance and structural stability than linear peptides. Tremendous efforts have been made towards developing of cell-permeable cyclic peptide drugs to block PPIs. Short CPP motifs have been rationally conjugated to otherwise cell-impermeable cyclic peptides to facilitate their intracellular uptake. This delivery strategy has been applied more generally to develop bicyclic peptide drugs comprised of one membrane-crossing CPP moiety and one cyclic peptide PPI inhibitor, while maintaining target specificity and affinity [52]. The oncogenic Ras-Raf interaction was blocked by a bicyclic peptide inhibitor that significantly impeded MEK/ATK signaling and led to apoptosis in lung cancer cells [53]. While CPPs are not themselves immunogenic, bioactive peptide-conjugated CPPs can sometimes induce an immune response, which may limit their application against some targets [54].

### 2.3. High-Throughput Screening (HTS) for New Peptide Leads

The discovery of the Ras-Raf bicyclic peptide inhibitor in fact stemmed from optimization of hits identified from a screen of 5.7 million bicyclic peptides for interaction with oncogenic K-Ras^G12V^. While high content combinatorial library screening method has facilitated the rapid identification of PPI inhibitors, peptides with weaker inhibitory activity may not be efficiently detected. Such modest interactions should not necessarily be ignored because sequence optimization or cyclization may be able to drastically improve affinity. The sequential rounds of “biopanning” enrichment in phage display library screening can improve the detection of weaker interactions. In fact the importance of this approach over the past three decades was recently recognized by the Nobel Prize for Chemistry award [55,56]. Over this period, phage display and recombinant DNA technologies have facilitated identification and optimization of new lead peptides against a diverse array of biological targets. The original approach involved sequential rounds of affinity enrichment and expansion, and finally identification of enriched phages. Although facilitating sensitive detection of weaker interactions, the high number of biopanning rounds involved can cause selection bias, dropouts and enrichment of false positives [57]. These issues have been much reduced by the recent application of next generation sequencing (NGS) analysis of phase display experiments. NGS is quantitative and sensitive enough to minimize the number of biopanning rounds needed to detect enriched interactions, minimizing the bias caused by multi-cycle screening. The low cycle number does however require that interactions bind fairly strongly [58]. Traditionally, phage-displayed libraries have been constrained by the necessity of using only linear display of non-modified naturally occurring amino acids. This limitation has recently been overcome by the development of strategies for on-phage chemical modifications including introduction of chemical entities such as cyclization linkers, fluorophores, small molecules or post-translational modifications like glycosylation. [59,60]. These advances in modern biopanning approaches support the notion that lead peptides of higher affinity and genuine bioactivity could be identified and then subjected to rational optimization of sequence and modifications for clinical development.

## 3. Peptides and Protein–Protein Interactions

PPIs are well-recognized potential therapeutic targets, given that dysregulated protein interaction networks underlie a wide range of pathologies. It is estimated that there are at least 140,000 pairwise PPIs in the human interactome [61]. Many efforts towards peptide innovations have been made to interfere with pathogenic PPIs to modulate the downstream signaling events. Such modulation with small molecules, however, has been difficult due to the large area of most larger PPI interfaces (generally 1500 Å^2^ to 3000 Å^2^), relative to the binding pocket size of the small molecules (300–1000 Å^2^) [62]. Small molecules generally do not interact with target proteins over a large enough surface area to block the interacting surface [63]. As discussed above, the physiochemical natures of peptides including the large and flexible backbones make them much better candidates in PPI inhibition than small molecules. Peptides that interfere with PPIs are termed interfering peptides (IPs) and are capable of binding to the larger grooves or clefts on an interacting face thus blocking that interaction surface. Another major advantage of IPs as the means of targeting PPIs over small molecules is the presence of amino acid residues that can interact with other residues at protein–protein interfaces [9]. With recent advances in techniques to overcome the intrinsic disadvantages of peptide drugs such as their limited stability, solubility and bioavailability, IPs as biotherapeutics are receiving increasing attention. In this section, we reviewed a number of promising successes in IP development against PPIs, as well as common strategies used to validate and optimize IPs as effective biotherapeutics.

### 3.1. Promising Developments for Interfering Peptides

Protein–protein interactions are at the core of normal and pathogenic cell biology and physiology. Abnormal protein–protein interactions drive signaling changes that underpin pathologies including infection, chronic inflammation, neurodegeneration, cancer and cardiovascular disease to name a few. Protein interaction surfaces are therefore attractive therapeutic targets, and as discussed peptides have advantages over small molecules in this area. A number of promising IPs are currently under clinical investigation. A 28-mer peptide drug that blocks the interaction of the ubiquitin-ligase MDM2 with its target p53 is able to block MDM2-dependent p53 ubiquitination, promoting p53 stabilization and tumor suppression [64,65]. A 17-mer peptide drug (CTCE-9908) is able to block CXCR4 activation in tumor cells, by blocking the interaction between CXCR4 and its ligand CXCL12. CTCE-9908 is under a phase I trial [66,67]. A peptide drug based on the N-terminal c-Jun sequence (XG-102, Brimapitide) competes with endogenous c-Jun for interaction with JNK, repressing JNK-driven inflammation. Brimapitide is under a phase III trial [68,69].

IPs with α-helical structures that bind to protein interacting surfaces have shown particularly promising interaction-blocking activity, probably due to their good stability and protease resistance. [70]. The literature has documented the effectiveness of α-helical peptides targeting several oncogenic protein interactions, including EZH2/PRC2, MDM2/p53, β-catenin/Wnt and Bax/Bcl-xL interactions. These structurally-modified peptide drugs are termed peptimimetics, and are designed to disrupt the flat and large interfaces of their respective targets. These cases demonstrate the therapeutic potential for peptide drugs to modulate pathogenic protein interactions with high specificity [71,72].

### 3.2. Experimental and Computational Methods for Determining PPI

Protein–protein interactions have been experimentally determined by a variety of biophysical techniques such as X-ray crystallography, NMR spectroscopy, surface plasma resonance (SPR), bio-layer interferometry (BLI), isothermal titration calorimetry (ITC), radio-ligand binding, spectrophotometric assays and fluorescence spectroscopy. Experimental data generated by these techniques has advanced our knowledge of how secondary and tertiary protein structure and interaction kinetics influence downstream biological events. These techniques, however, are often time-consuming and applied to study one specific PPI at a time. While protein crystal X-ray diffraction is undoubtedly a very powerful structural analysis approach, which is capable of defining structure down to individual atoms, it has major technical challenges. Many proteins do not crystalize well, or do not crystalize except as smaller protein domains. Even if each protein in a complex forms crystals alone, co-crystallization can be especially challenging. NMR spectroscopy can generate protein complex structures, but has a lower resolution compared to X-ray diffraction. Optical or calorimetric approaches can provide information about the energy, affinity and disassociation properties of an interaction, but do not identify a specific interaction surface the way NMR or X-ray diffraction do. The technical challenges and poor scalability of wet-lab experimental approaches has necessitated the development of reliable computational strategies. Computational docking methods have thus been developed to expedite the process of generating accurate predictions of protein structure, surface charge and interaction affinities. Even for proteins without an available crystal structure, these programs can be applied for in silico identification of key protein interaction surfaces, the structural effect of mutations and binding analysis of potential blocking peptides.

#### 3.2.1. Computational Docking Strategies

Computational PPI docking has provided rapid and useful information for drug designs at the atomic level, as some of the docking methods can be executed within the order of minutes. This can be achieved by rigid-body docking strategies, in which two interacting proteins are both considered absolutely rigid in calculation for optimization of chemical and geometric orientation fit. Z-DOCK is a typical rigid-body protein docking program that generally generates accurate predictions of PPI when proper scoring scaffolds are provided [73]. Due to the availability and complexity of various scoring parameters from most flexible docking methods, a variety of different docking programs have been developed over the past decades. ATTRACT, for instance, is a well-established PPI prediction server with powerful toolkits covering many scoring parameters but is less user-friendly [74].

#### 3.2.2. Sequence- or Structural-based Predictions

Many computational docking strategies, especially flexible-body docking methods, require structural information including number of hydrogen bonds, buried surface area, mutation hotspots, geometric angles and allosteric effects for calculations of binding free energies to generate more accurate predictions on binding affinities between the interacting proteins [75]. By contrast, sequence-based strategies rely on the sequence and functional information in many publicly available databases to generate predictions of binding affinity. PPA-Pred, for example, developed a model based on sequence features by classifying protein–protein complexes according to their biological functions and percentage of binding residues for binding affinity prediction [76]. Despite offering less confident prediction on binding affinity and an inability to predict conformational binding poses, sequence-based models can be refined with dataset updates in experimental and functional scaffolds. In fact, sequence-based strategies also utilize learning machines to enhance their prediction confidence over time [77].

Although significant progress has been made in scoring functions from both strategies, the lack of high computing power and larger experimental datasets with high quality have restricted the advances in the field. A community-wide experiment CAPRI compared computationally predicted protein complex structures with experimentally determined structures to evaluate and rank the best-performing servers based on prediction accuracy [78]. HADDOCK and ClusPro are ranked the top prediction servers that use rigid-body docking methods based on root mean square deviation (RMSD) to yield binding free energy and buried surface area at highest confidence [79,80,81].

## 4. Innovations and Computational Methods for Peptide—Protein Interactions

Similar to protein–protein interactions, structural information (either a sole target protein structure or complex structure with ligand) that is available for a drug target has often limited prediction accuracy for PepPIs. As a result of the lack of protein co-structures, many studies utilize existing information from structural databases such as the Protein Data Bank (PDB) to identify sequence-binding motifs for peptide designs. [82]. Another database PepX is comprised of more than 500 experimentally studied peptide interactions with high-resolution structures and allows simple inputs of user-defined peptide templates [83]. In silica mutation hotspot analyses of protein–peptide interfaces suggested 6–11 amino acid long peptides usually contain 2–3 residues that form critical contacts with the target protein. Despite its apparent similarity to modeling protein–protein interactions, PepPI analyses can be surprisingly complex due to the diverse structural changes that could arise from flexible side-chains and backbones within a peptide [84,85]. Short peptides of up to about 15 residues usually form simpler α-helix or β-sheet structures, the structures of longer peptides are more difficult to predict due to their backbone rearrangements. The degree of complexity in peptide structure prediction further increases as the flexibility of target protein conformation is considered [86]. In this section, we will review recent computational models that are developed to overcome these challenges for more reliable peptide drug designs against PPIs. Selected PepPI prediction methods and summaries of their key features discussed in this section are provided as an overview in Table 2.

### 4.1. Selection of Initial Peptide Scaffolds

Before discussing current computational methods for PepPIs, we would like to first introduce recent advances in the selection of initial peptide scaffolds that also play critical roles in peptide drug development. A number of well-characterized natural occurring peptides had been selected from natural proteins and demonstrated to preserve original functions including structural scaffolds or the ability of recognizing target molecule. Repeated Arg-Gly-Asp (RGD) motifs, for instance, were first derived from cell attachment domain of fibronectin that binds to membrane-bound receptor protein integrins and activates cellular growth, differentiation, adhesion and migration [87]. The capability of RGD peptides in mimicking the functions of their natural protein has served as a promising strategy for not only therapeutic PPI interferences but also structural and functional analyses of proteins. In addition to the above-mentioned chemical and phage peptide libraries, in silica modeling-based design (sections below) has recently emerged as a powerful approach to new peptidic leads identification from natural proteins. Another interesting development is the identification of microtubule-binding peptides. Microtubules are hollow tubular protein assemblies composed of intracellular α-/β- tubulin dimers with significant nanodevice implications attributed to their involvement in many eukaryotic cell functions including tumor progression. Peptide-modulated nanodevice-encapsulating drugs targeting intracellular tubulins under different formulations such as peptide-conjugating liposomes or peptide-drug assemblies to exert synergistic anti-cancer effects have attracted vast attentions [88,89]. A recent pioneer study further demonstrated that peptides selected from microtubule-associated protein Tau functionalized inner surface of microtubule by encapsulation of gold nanoparticles inside microtubules [90]. Moreover, another intriguing discovery of a tetrapeptide Ser-Leu-Arg-Pro (SLRP) from a peptide library was shown to perturb microtubule function and lead to apoptosis of cancer cells [91]. Of note, the selection of SLRP was assisted by the computation docking method Autodock Vina.

### 4.2. Docking Peptide–Protein Interactions

Successful docking of the structural pose of a PepPI has been largely dependent on the extent of structural scaffolds available about the interaction complex. The dramatic increases in numbers of peptide–protein structures available in PDB have greatly facilitated the development of more powerful docking and refinement methods in predicting accurate PepPIs. Peptide–protein docking strategies are usually categorized into local or global docking based on the extent of structural information provided as inputs.

#### 4.2.1. Local and Global Docking Methods

Local docking is the mostly commonly used strategy as it searches for a potential binding pose for peptide at a user-defined binding site in a resolved structure of its target receptor. A number of methods have the capability to improve initial model quality at atomic resolution within 1 Å to 2 Å RMSD of the experimental peptide conformation. DynaRock, Rosetta FlexPepDock and PepCrawler are the most popular methods that provide different approaches of defining peptide-binding sites. DynaDock uses soft-core potential combined with Molecular Dynamics as refinement approach for conformational sampling and receptor side-chain flexibility determination [92]. As van der Waals and Coulomb energy potentials were smoothened in this protocol, faster conformational sampling of the peptide–protein complex was achieved when the soft-core potential gradually converged to a physical potential as the simulation progressed. Rosetta FlexPepDock is a Monte Carlo-based method that minimizes optimization steps to yield high-quality conformational sampling for well-characterized binding motifs with hotspot residues [93,94]. This protocol was validated against a large dataset with rigid-body sample docking and variable degrees of backbone modeling. PepCrawler utilizes an algorithmic robotics motion planning called Rapidly-exploring Random Tree (RRT) to optimize peptide structural poses at binding sites [95]. In this refinement protocol, a conformation tree for the peptide–protein complex is built for the resulting models that are automatically clustered by local shape analysis of energy funnel.

However, not every query peptide has available information of backbone conformation. Sampling methods that allow acquisition of near-native peptide conformation become essential prior to performing local docking. Rosetta FlexPepDock ab initio, for instance, is a protocol that combines ab initio peptide folding with local docking by placing the query peptide into a user-defined binding site from any arbitrary backbone conformation [96]. The binding site can be defined through positioning of a hotspot residue (with side-chain) or using the standard constrains for binding sites provided by Rosetta FlexPepDock. Recently, HADDOCK method (HADDOCK peptide docking) was used to hypothesize the unnecessity of prior backbone information in local docking using secondary structure: α-helix, extended or polyproline-II helix as an ensemble of canonical conformation constrained to a defined binding site [97]. Further, a number of small molecule docking methods such as Gold, Surflex and AutoDock Vina have been applied to perform local docking for short peptides of less than five amino acids [98,99,100]. Despite sub-optimal near-native modeling results were obtained, an interesting docking protocol DINC 2.0 proposed to overcome the limitation by docking peptide fragments [101].

#### 4.2.2. Global Docking Methods

Unlike local docking that searches for the peptide-binding pose, global docking methods also search for the peptide-binding site at the target protein. Global docking is often the method of choice when no prior information is available on binding sites. A spatial position specific scoring matrix (PSSM) was used in developing the PepSite method to identify potential binding sites with estimated position for each residue [102,103]. The variable degrees of peptide backbone/side-chain flexibility, nonetheless, render flexible-body docking extremely inefficient. Standard procedures for global peptide–protein docking thus usually depend on rigid-body docking following acquisition of input peptide conformation. Several global docking methods are capable of predicting peptide conformation from a given query sequence. ClusPro (ClusPro PeptiDock) and ATTRACT (pepATTRACT) for example, use a pre-defined motif set of template conformations for threading query sequences. The generated peptide conformations are next rigid-body docked in one simulation round [104,105]. Other global docking methods such as PeptiMap, AnchorDock and CABS-Dock also provide automatic docking simulation with varying algorithms such as small molecule binding adaption, in-solvent simulation, flexibility of query peptide or target protein at predicted binding proximity [106,107,108].

In addition to highly accurate predictions made by PIPER-FlexPepDock, another recently developed method HPEPDOCK used an ensemble of peptide conformations for blind global docking and obtained significantly higher success rates as well as lower simulation time required than pepATTRACT [105,109].

#### 4.2.3. Template-Based Docking Method

Template-based docking methods are also known as comparative docking strategies. They use known structures as template scaffolds by threading the sequence of the query peptide and/or target protein to build a model of the interacting complex [78]. Template-based docking has recently been considered as a new category in peptide–protein docking due to the rapid increases in the number of peptide–protein structures deposited in PBD, which have greatly expedited advances and designs in simulation algorithms. The GalaxyPepDock is a popular server that performs such similarity-based docking by searching templates of highest similarity and builds models using energy optimization to allow more accurate predictions on structural flexibility between interacting complexes [110]. GalaxyPepDock demonstrated superior prediction results than other servers using PeptiDB datasets during CAPRI blind prediction experiments. Recently, a machine learning (Rain Forest) based method SPRINT-Str used experimental structural information for robust and consistent prediction on peptide–protein binding residues and sites [111]. Another template- and machine learning-based docking method, PBRpredict, utilized models trained from peptide-binding residues of diverse types of domains to build models that robustly predict interacting residues in peptide-binding domains from target protein sequences [112]. The use of computational machine learning algorithms is reminiscent to the prediction servers for CPP and has become commonly utilized in optimization of clustering and scoring in methods for predicting PepPIs. A commonly accessible online program for computational design of peptide–protein, PepComposer, also implemented a machine learning algorithm (Monte Carlo) in Pyrosetta for a fully automatic computational peptide design that was demonstrated to predict known PepPIs at highly reproducible rates [113].

## 5. Concluding Remarks

Peptides have attracted a lot of attention and the number of approved peptide biotherapeutics has been on the incline over the recent decades. This has been an attractive approach due to their ability to bind with larger interfacial pockets than small molecules, and has been driven by major advances in computational structural prediction and the expansion of available chemical modifications to improve stability, affinity and specificity. The publicly available computational binding prediction tools have led to effective rational designs of new peptide drugs of higher therapeutic potency. Our recent study utilized both biological and computational methods to develop a cancer-specific targeting peptide that facilitated significantly greater in vitro and in vivo and therapeutic efficacy [114].

**Figure 1:**
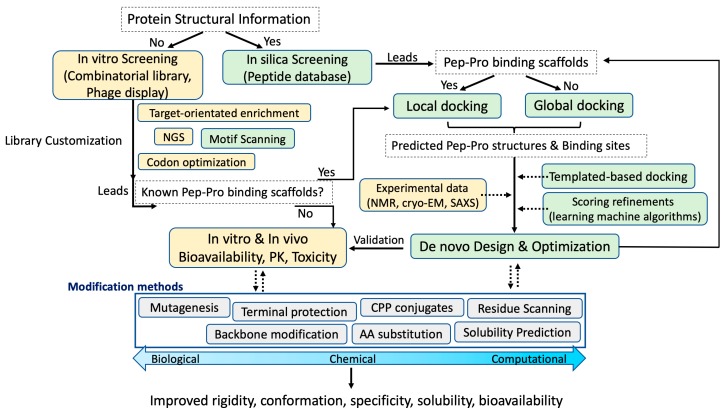
A modular view of the peptide drug development cycle. This flowchart provides a summarized overview for topics covered in this review. Boxes in green color indicate computational methods; gold are biological methods; grey are commonly modification methods applied for improving peptide bioactivity. The blue two-headed arrow represents the modification methods that are relatively more biological, chemical or computational. White dashed boxes are criteria for accessing which method can be chosen next depending on availability of information. Solid or dashed arrows indicate direct or optional connections between methods, respectively.

Despite recent advances in computational modeling of protein–protein and peptide–protein structures, major challenges remain. For instance, it remains challenging to simultaneously consider the backbone and side-chain flexibility of the peptide and its target protein to accurately predict the bound structure. Secondly, interpretation of experimental data from high-resolution NMR spectroscopy and cryo-electromicroscopy or small-angle X-ray scattering (SAXS) to obtain accurate experimental structures is often ambiguous, which makes integration of such experimental data into computational prediction software very difficult. There are computational servers that have attempted to translate ambiguous experimental data into algorithmic constrains that can be applied as an option for docking [79,97]. In fact, those docking methods become quite helpful for experimental biologists as a means of validating the proposed binding mechanism. Thirdly, scoring had also been a great challenge because many lower-ranked models were found to be of higher quality in the docking results and vice versa. It was attributed to most scoring were solely based on binding energy for clustering. Recently, CAPRI experiments revealed that a hybrid model selection methodology of utilizing both energy-based scoring as well as other methods such as mutagenesis, co-evolutionary information, sequence- or structural-clustering function could generate accurate peptide–protein docking results that are closer to native models [78,80,115]. In this review, we summarized various works from the fields of biology, chemistry and computation that are relevant to peptide drug development. Increasing interests in peptide biotherapeutics has sparked rapid advances in both chemical and biocomputational methods. Figure 1 provides a modular view for a common peptide drug development cycle that covers the diverse topics discussed. Although this figure may not cover every modern technique used in peptide drug development to date, the generalized concepts and workflow emphasize that neither the biological, chemical nor computational method is indispensable for greater peptide drug discovery and development. We also anticipate that peptide–protein docking methods will become more commonly used tools to support experimental work for better structural-based peptide drug design and discovery.

## Figures and Tables

**Table 1:** Overview of prediction methods for peptide solubility and cell-penetrating peptides.

Method	Learning Machine Model	Input Length (aa)	Input Format	Multiple Entry	Database	Web Server	Refs
**Peptide Solubility**							
ccSOL omics	Super vector machine (SVM)	–	FASTA	Yes (up to 10^4^)	Target Track (non-redundant) (http://sbkb.org/tt/)	http://s.tartaglialab.com/static_files/shared/tutorial_ccsol_omics.html	[39]
PROSO II	Super vector machine (SVM)	21 to 2000	FASTA	Yes (up to 50)	Target Track (http://sbkb.org/tt/)	http://mbiljj45.bio.med.uni-muenchen.de:8888/prosoII/prosoII.seam	[40]
**Cell-Penetrating Peptides**							
CPPpred	Artificial neural networks (ANN)	5 to 30	FASTA	Yes	CPPsite	http://bioware.ucd.ie/cpppred	[47]
CPPpred-RF	Random forest (RF)	–	FASTA	Yes	CPP924 and CPPsite3	http://server.malab.cn/CPPred-RF	[46]
KELM-CPPpred	Kernel extreme learning model (KELM)	5 to 30	FASTA	Yes	Curated 408 CPP/non-CPP	http://sairam.people.iitgn.ac.in/KELM-CPPpred.html	[48]
CellPPD	Super vector machine (SVM)	–	FASTA	Yes	CPPsite1,2,3	http://crdd.osdd.net/raghava/cellppd/multi_pep.php	[50]
CPPsite 2.0	–	–	FASTA	Yes	1855 uniquely curated	http://crdd.osdd.net/raghava/cppsite/	[51]

**Table 2:** Summary for peptide–protein interactions docking methods.

Methods	Key Features	Model Quality ^#^	Web Server	Refs
**Local Docking**				
DynaDock	▪ Combined Optimized Potential Molecular dynamics (OPMD) with a soft-core potential ▪ Faster conformational sampling ▪ Smoothened van der Waals and Coulomb energy potentials ▪ Full flexibility of peptide and target protein	Near-native	Not available to public	[92]
Rosetta FlexPepDock	▪ Monte Carlo-based optimization ▪ High-quality conformational sampling ▪ Hotspot residue (side-chain) modeling ▪ Receptor flexibility (side-chains to full structure) ▪ Rosetta energy function based clustering and scoring	Sub-angstrom *	http://flexpepdock.furmanlab.cs.huji.ac.il or http://www.rosettacommons.org/software	[93]
PepCrawler	▪ Rapidly-exploring Random Tree (RRT) algorithm ▪ Motion-planning based sampling ▪ Ranking by automated energy funnel analysis (clustering-based) ▪ Fully flexible peptide structures	Near-native *	http://bioinfo3d.cs.tau.ac.il/PepCrawler	[95]
Rosetta FlexPepDock *ab initio*	▪ Ab initio modeling based on Rosetta fragment library ▪ Simultaneous docking and de-novo folding of peptides ▪ Peptide secondary structure option ▪ No information for peptide conformation required	Near-native to Sub-angstrom ^§^	http://www.rosettacommons.org/software	[96]
HADDOCK peptide docking	▪ Modeling from ensemble of three canonical secondary structures (α-helix, extended or polyproline-II helix) ▪ User-defined residues at binding pocket ▪ Binding free energy based scoring ▪ Fully flexible for interacting residues of peptide and protein	Near-native *	http://haddock.science.uu.nl/services/HADDOCK2.2/	[79,97]
PepSite 2.0	▪ Identifies most peptide-binding site in seconds ▪ Generates low-resolution model of peptide ▪ Coarse-grained peptide orientation by spatial position-specific scoring matrix (S-PSSM)	Medium ^†^	http://pepsite2.russelllab.org	[102]
**Global Docking**				
ClusPro PeptiDock	▪ Fast Fourier Transform (FFT)-based docking method ▪ Motif-based prediction for peptide conformation ▪ Clustering by structure scoring and CAPRI peptide docking criteria	Near-native to Sub-angstrom ^§^	https://peptidock.cluspro.org/	[81,104]
pepATTRACT	▪ Peptide structure prediction by threading sequence onto the three peptide conformations (as HADDOCK peptide docking) ▪ Rigid-body peptide docking within binding pocket ▪ Suitable for large-scale in silico protein–peptide docking ▪ Clustering based on ATTRACT scores ▪ Optional flexible docking for interacting residues	Near-native to Sub-angstrom ^§^	http://bioserv.rpbs.univ-paris-diderot.fr/services/pepATTRACT/	[105]
HPEPDOCK	▪ Hierarchical algorithm ▪ Ensemble peptide conformation by MODPEP ▪ Blind global peptide docking ▪ Higher success rate and lower processing time for both global and local docking	Near-native to Sub-angstrom ^§^	http://huanglab.phys.hust.edu.cn/hpepdock/	[109]
**Template-based**				
GalaxyPepDock	▪ Use similarity search (known template structures) as scaffolds for prediction ▪ Energy-based model optimization and scoring ▪ Superior accuracy using PeptiDB datasets than other servers	Medium (ligand); Near-native (interface)	http://galaxy.seoklab.org/pepdock	[110]
SPRINT-Str	▪ Predict residues at peptide–protein binding interface ▪ Use SVM with optimized parameters ▪ Capability to distinguish binding sites of peptide from DNA, RNA and carbohydrate	N/A	http://sparks-lab.org/server/SPRINT-Str	[111]
PBRpredict-Suite	▪ Predict interacting residues based on peptide-binding domain (PDB) from template sequences in NCBI database ▪ Integrated six machine learning algorithms (model stacking) ▪ Proteome-wide prediction feasibility	N/A	http://cs.uno.edu/~tamjid/Software/PBRpredict/pbrpredict-suite.zip	[112]
PepComposer	▪ Motif similarity search to defined binding interfaces from monomeric protein databases PepX (http://pepx.switchlab.org) ▪ Monte carlo-implemented PyRosetta ▪ User-defined options for binding site residues or chain selection	Near-native	https://cassandra.med.uniroma1.it/pepcomposer/webserver/pepcomposer.php	[113]

^#^ RMSD of peptide backbone to experimental structure data. Medium: Between 2 Å to 5 Å; Near-native: 1 Å to 2 Å; Sub-angstrom: Less than 1 Å. * Tested on PeptiDB dataset. ^†^ Customized dataset of 405 known protein–peptide complexes with unbound receptor models. ^§^ On selected subsets of PeptiDB.
